# Genetic Diversity and Selection Footprints in the Genome of Brazilian Soybean Cultivars

**DOI:** 10.3389/fpls.2022.842571

**Published:** 2022-03-30

**Authors:** Heitor Calux Mendonça, Luiz Filipe Protasio Pereira, João Vitor Maldonado dos Santos, Anderson Rotter Meda, Gustavo César Sant’ Ana

**Affiliations:** ^1^Centro de Ciências Biológicas, State University of Londrina, Londrina, Brazil; ^2^Laboratório de Biotecnologia, Instituto de Desenvolvimento Rural do Paraná, Embrapa Café, Londrina, Brazil; ^3^Tropical Melhoramento e Genética (TMG), Cambé, Brazil

**Keywords:** Brazilian Soybean, adaptation, selection signatures in the genome, population structure, genotyping by sequencing

## Abstract

Although Brazil is currently the largest soybean producer in the world, only a small number of studies have analyzed the genetic diversity of Brazilian soybean. These studies have shown the existence of a narrow genetic base. The objectives of this work were to analyze the population structure and genetic diversity, and to identify selection signatures in the genome of soybean germplasms from different companies in Brazil. A panel consisting of 343 soybean lines from Brazil, North America, and Asia was genotyped using genotyping by sequencing (GBS). Population structure was assessed by Bayesian and multivariate approaches. Genetic diversity was analyzed using metrics such as the fixation index, nucleotide diversity, genetic dissimilarity, and linkage disequilibrium. The software BayeScan was used to detect selection signatures between Brazilian and Asian accessions as well as among Brazilian germplasms. Region of origin, company of origin, and relative maturity group (RMG) all had a significant influence on population structure. Varieties belonging to the same company and especially to the same RMG exhibited a high level of genetic similarity. This result was exacerbated among early maturing accessions. Brazilian soybean showed significantly lower genetic diversity when compared to Asian accessions. This was expected, because the crop’s region of origin is its main genetic diversity reserve. We identified 7 genomic regions under selection between the Brazilian and Asian accessions, and 27 among Brazilian varieties developed by different companies. Associated with these genomic regions, we found 96 quantitative trait loci (QTLs) for important soybean breeding traits such as flowering, maturity, plant architecture, productivity components, pathogen resistance, and seed composition. Some of the QTLs associated with the markers under selection have genes of great importance to soybean’s regional adaptation. The results reported herein allowed to expand the knowledge about the organization of the genetic variability of the Brazilian soybean germplasm. Furthermore, it was possible to identify genomic regions under selection possibly associated with the adaptation of soybean to Brazilian environments.

## Introduction

Soybean [*Glycine max* (L.) Merril] is one of the crops of most economic importance in the world. Brazil is currently the largest producer in the world, responsible for nearly 40% of the global soybean supply (FAOSTAT, 2019; Instituto Brasileiro de Geografia e Estatística [IBGE], 2021). This Brazilian leadership in world soybean production has been possible through the efficient exploration of genetic diversity by different breeding programs. Thus, a better understanding of how to preserve and increase this diversity as well as identify selection signatures in the soybean genome associated with its adaptation to Brazilian growing environments is paramount to allowing further genetic gains in yield.

Although the genetic diversity present in soybean germplasms all over the world have been studied extensively, in Brazil, these studies have been scarce. Brazilian germplasms present a particularly narrow genetic base when compared to Chinese, European, and North American breeding programs (Priolli et al., 2013; Gwinner et al., 2017; Liu et al., 2017; Žulj Mihaljević et al., 2020). In their seminal paper of 1986, Hiromoto and Vello (1986) found that the genetic base of Brazilian soybean largely came from only 26 common ancestors, with only 4 (CNS, Tokyo, Roanoke, and S-100) contributing approximately half of this base. The genetic diversity of soybean germplasms in Brazilian breeding programs stayed relatively stable from 1970 to 2000 (Priolli et al., 2004). More recently, a study of the genetic base of 444 Brazilian cultivars found that while the number of common ancestors had risen to 60, a small subset of these were responsible for most of the genetic base (Wysmierski and Vello, 2013).

Crop domestication and founding events contribute to a reduction in the available genetic diversity of several crops (Hyten et al., 2006; Liu et al., 2019). Soybean was domesticated approximately 5,000 years ago in China (Xu et al., 2002; Guo et al., 2010; Li et al., 2010; Han et al., 2016; Wang J. et al., 2016; Jeong et al., 2019) and has since been introduced to countries all over the world. Soybean was introduced in North America and Europe in the nineteenth century, and until approximately 1940, most soybean varieties grown in the United States were introduced materials. Soybean breeding programs then began producing cultivars adapted to North American growing environments, establishing the North American soybean’s genetic base (Carter et al., 2004).

Around 20 years after the United States started its breeding programs, a small number of North American cultivars were brought from the United States to Brazil. Breeders started crossing these materials among themselves and with other sources to generate cultivars adapted to the growing environments in Brazil. This sequence of founding events formed genetic diversity bottlenecks that culminated with the narrow genetic base now seen in Brazilian soybean germplasms (Miranda et al., 2007; Contreras-Soto et al., 2017a; Gwinner et al., 2017). An increase in the genetic diversity available to breeders could be achieved by using genotypes from different origins that have a high level of genetic dissimilarity or divergence (de Almeida et al., 1999). Thus, a quantitative analysis of the genetic dissimilarity between pairs or potential parentals becomes an important resource for breeding programs, allowing for a more efficient choice of parentals to generate progenies with high genetic variance (Vieira et al., 2005; de Almeida et al., 2012), which could potentially increase the selection gain.

While founding events and domestication cause a decrease in the genetic diversity of a crop, this reduction isn’t uniform throughout the entire genome. Genomic regions associated with traits of agronomic importance and adaptation to the environment are invariably under greater selection pressure. Thus, events such as domestication as well as artificial selection by breeding programs can cause a sharp reduction in the genetic diversity in specific parts of a crop’s genome (Maynard Smith and Haigh, 1974; Vigouroux et al., 2002). These signatures of selection have been used to study several crops’ domestication and breeding history and to direct introgression efforts aimed at increasing a germplasms’ genetic diversity at specific genomic regions (Tanksley and McCouch, 1997; Jun et al., 2011; Ge et al., 2012; Wang J. et al., 2016; Grainger et al., 2018; Saleem et al., 2021; Zhang et al., 2021).

The last decade has seen the advent of techniques that combine next generation sequencers with multiplex sequencing approaches to allow for the high throughput genotyping of large diversity panels at a relatively low cost. Approaches such as genotyping by sequencing (GBS) (Elshire et al., 2011) greatly reduced genotyping costs by focusing the sequencing and variant calling on euchromatic genomic regions with a high concentration of coding sequences, which makes it a useful tool for studying the genetic diversity present in soybean germplasms (Bruce et al., 2019).

We used GBS to assess a diverse collection of soybean accessions from different breeding companies and geographic regions of origin to (1) investigate the population structure and genetic diversity among soybean cultivars from different Brazilian breeding programs, (2) identify genomic regions under selection among cultivars developed by different companies as well as between different geographic regions, and (3) identify quantitative trait loci (QTLs) present in those genomic regions.

## Materials and Methods

### Plant Materials and Single Nucleotide Polymorphism Genotyping

A panel consisting of 343 soybean accessions was used in this study. Of those, 263 were Brazilian cultivars developed by several different companies in the last two decades, 54 were accessions collected on the Asian continent in the twentieth century, and 26 were North American genotypes developed between 1956 and 1995 (Supplementary Table 1).

Genomic DNA was extracted from leaf tissue from a single plant per genotype using the DNA-easy Plant Kit (Qiagen) following the manufacturer protocols. The GBS libraries were constructed at the Plate-Forme d’Analyses Génomiques (Université Laval, Québec, Canada) following a protocol established by Elshire et al. (2011) using the *Ape*KI restriction enzyme. Library sequencing was performed with the Illumina HiSeq2000 system.

Briefly, after demultiplexing was performed based on barcodes, a sample of 1,000,000 *reads* was used for quality control (QC) using the FastQC platform. Reads with invalid barcodes or containing adapter or primer sequences were filtered. Furthermore, low quality bases (PHRED score < 15) were removed from the 3’-end of each sequence; reads shorter than 20 bp at the end of this process were also filtered.

Single nucleotide polymorphism (SNP) calling was performed using Platypus (Rimmer et al., 2014) and bcftools (Li, 2011) on sequences aligned to the soybean reference genome Gmax 2.0^1^ using the Bowtie package (Langmead and Salzberg, 2012). The parameters used in both pipelines were similar. In brief, to be considered in the Platypus pipeline, SNPs needed to present a PHRED score higher than 15 and have a sequencing coverage value higher than 5 × in at least 20% of the reads that contained the polymorphism. In the bcftools pipeline, the minimum QUAL and PHRED scores were maintained at 15, while minimum coverage was set to 15×.

The resulting set of markers underwent further QC using TASSEL software (Bradbury et al., 2007). The SNPs with a minor allele frequency less than 5%, call rate below 75%, and heterozygosity higher than 50% were filtered. Subsequently, missing data was imputed using the LD-kNNi method (Money et al., 2015).

### Population Structure and Genetic Diversity Analysis

Population structure was assessed by Bayesian and multivariate approaches. We performed a population structure analysis using a model-based Bayesian approach in Structure v.2.3.4 (Pritchard et al., 2000) through the *Structure_threader* plug-in (Pina-Martins et al., 2017). The SNPs used in the analysis were filtered based on linkage disequilibrium (LD; *R*^2^< 0.25) using PLINK software (Purcell et al., 2007), which resulted in a set of 959 SNPs. The *K*-values varied from 1 to 8, with 10 runs for each *K*-value. Burn-in time and replication number were set to 50,000 and 75,000, respectively. The *K*-values that best fit the data were determined using the *Evanno* approach (Evanno et al., 2005). This method uses a Δ*K-*value to compare variations in the log probability of the data for successive *K*-values. Population stratification was also explored using a principal component analysis in the TASSEL software (Bradbury et al., 2007).

An LD decay analysis was performed using PLINK (Purcell et al., 2007) based on 2,175 SNPs. Average LD decay was computed for each chromosome using the *R*^2^-value for all pairwise comparisons between SNPs in 1 Mb windows. This analysis was conducted for the Brazilian set of cultivars as well as for the Asian set of accessions. To visualize the average LD decay profile, a cubic spline regression approach was adopted using the ggplot2 package in R (Wickham, 2016). Genome-wide LD decay was estimated using the spline method with the LD output from all chromosomes.

Diversity estimates were computed for the Brazilian and Asian accessions separately. The nucleotide diversity index (π) and fixation indexes (*F*_*ST*_) were calculated for each SNP site using VCFtools v0.1.16 (Danecek et al., 2011). Genetic distance among Brazilian cultivars was calculated using the IBS (Identity by State) metric in TASSEL. The genetic distances were used in the construction of a dendrogram using the UPGMA (unweighted pair group method with arithmetic mean) algorithm. The grouping pattern was then analyzed as a function of RMG and company of origin to better understand the influence of those factors on population stratification.

### Identification of Selection Signatures in the Soybean Genome

To detect selection footprints between the Brazilian and Asian accessions as well as among different Brazilian companies’ germplasms, we used an outlier locus detection approach through BayeScan (Foll and Gaggiotti, 2008). We performed 20 pilot runs of 5,000 iterations each, followed by 550,000 iterations on a sample size of 50,000 with a thinning interval of 10. The prior odds were set to 10, and SNPs with a *q*-value < 0.1 were considered statistically significant.

### Identification of Quantitative Trait Loci Present in Genomic Regions Under Selection

To identify QTLs located in genomic regions under selection, we used soybean QTL data from the Soybase database (see text footnote 1). All SNPs under selection as detected by BayeScan were mapped to the soybean reference genome. A SNP was within a QTL’s genomic region if its distance to the QTL was shorter than the average LD decay distance previously defined for the panel.

## Results

### Genotyping by Sequencing

The SNP calling by Platypus and bcftools identified 346,919 and 498,931 polymorphisms, respectively. Upon applying the QC steps, 33,359 and 24,865 SNPs were left, of which 12,757 were identified by both pipelines. Further filtering by TASSEL software resulted in a set of 2,175 high quality markers that were used in the subsequent analyses.

The SNP distribution throughout the genome was not uniform. Heterochromatic regions presented a markedly lower number of markers, with 87% of the sequenced polymorphisms concentrated in euchromatic regions. On average, 109 SNPs were identified per chromosome. Chromosome 18 harbored the highest number of SNPs (231), while chromosome 12 had the lowest number (68).

As expected from highly endogamic lines, on average, the SNPs presented with low heterozygosity (5.47%). The average minor allele frequency value after QC was 0.171, with a median of 0.136 and standard deviation of 0.115. Among the SNPs sequenced in this panel, 59.8% were transitions, and 40.2% were transversions.

### Population Structure and Genetic Diversity

**FIGURE 1:**
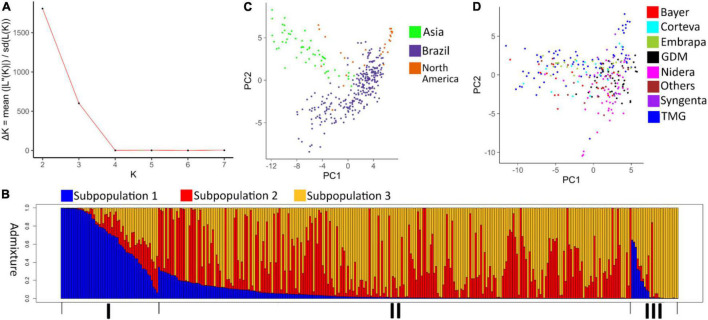
**(A)** Delta *K*-values for *k* = 1–8. **(B)** Analysis of the population structure using 343 soybean accessions with *K* = 3. (I) corresponds to the group of soybeans of Asian origin; (II) formed by Brazilian accessions; (III) formed by North American accessions. **(C)** Principal component analysis of 343 soybean accessions classified by geographic origin. **(D)** Principal component analysis of 247 Brazilian soybean cultivars classified by company of origin.

The best *K*-values chosen by the Evanno method were 2 and 3 (Figure 1A). Given the accessions’ geographic regions of origin, *K* = 3 was further explored. Each subpopulation concentrated individuals from a different geographic region (Figure 1B). Subpopulation 1 was formed mostly by Asian genotypes. Subpopulation 2 corresponded mainly to late maturing Brazilian genotypes and a single North American genotype. Subpopulation 3 concentrated most of the North American genotypes and early maturing Brazilian genotypes.

The population structure was also assessed by a principal component analysis. Using the first 2 principal components (explaining 7.6 and 3.8% of the total variation, respectively) as a basis, the genotypes’ distribution indicated the formation of 3 clusters, each formed primarily by accessions from a different geographic region (Figure 1C). A principal component analysis was also used to assess the stratification and diversity among Brazilian genotypes from different companies. In this analysis, the first two components explained 7 and 3.1% of the total variation. Cultivars from companies such TMG and Nidera showed a wider genetic base compared to companies such as GDM and Syngenta (Figure 1D).

A smaller average *F*_*ST*_ value was observed among Brazilian genotypes from different companies (0.0417), while a larger value was obtained when comparing Brazilian genotypes to Asian genotypes (0.102). This indicates a high level of genetic similarity among varieties developed by Brazilian companies, especially when compared to Asian genotypes. Average π among Brazilian genotypes was markedly lower than that for the Asian genotypes, 2.53 × 10^–6^ vs. 3.00 × 10^–6^.

**FIGURE 2:**
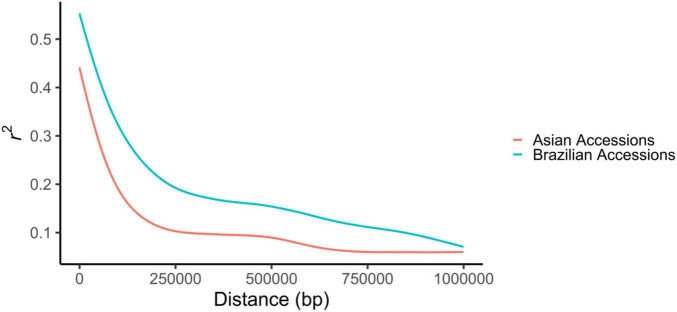
Linkage disequilibrium decay among Brazilian and Asian accessions.

The LD decayed as a function of the physical distance between markers in both the Brazilian and Asian genotypes. The average *R*^2^ was 0.237 among Brazilian genotypes and 0.161 among Asian genotypes. The distance over which the LD decayed to below 0.2 was around 230 kb in the Brazilian genotypes, whereas in the Asian genotypes, this value was much lower, only 94 kb (Figure 2).

We observed two grouping patterns. First, cultivars belonging to the same RMG grouped together, with early maturing cultivars presenting a higher degree of genetic similarity among themselves. Second, materials developed by the same company showed higher levels of similarity, which drove the grouping pattern (Supplementary Figure 1).

### Identification of Selection Signatures in the Soybean Genome

**FIGURE 3:**
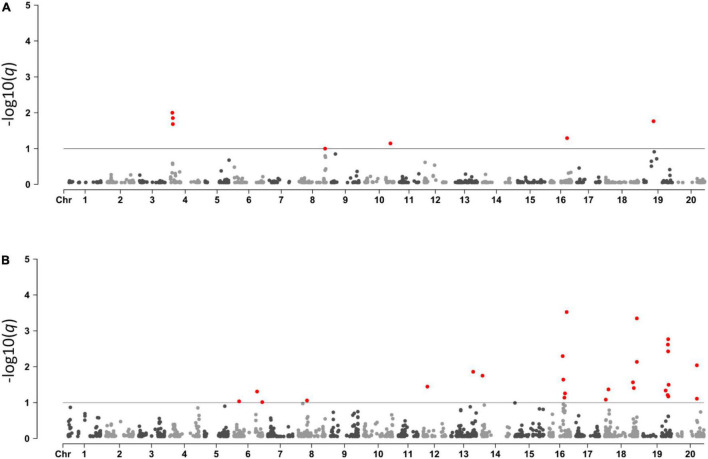
Manhattan plots showing SNPs under selection between Brazilian and Asian lines **(A)** and among different Brazilian breeding programs **(B)**.

BayeScan was used to detect selection signatures in the Brazilian × Asian genotypes and among Brazilian genotypes from different companies. In the Brazilian × Asian genotypes, we used 2,099 SNPs and 317 accessions, of which 263 were Brazilian cultivars and 54 were Asian genotypes. We identified 7 SNPs under selection in five different chromosomes, 4, 8, 10, 16, and 19 (Figure 3A and Supplementary Table 2). All SNPs except for SNP 1.7 were fixed among the Brazilian genotypes. In the second analysis, 260 Brazilian cultivars from 10 different companies and 1,850 SNPs were used. In total, 27 SNPs under selection in 9 different chromosomes were identified (Figure 3B and Supplementary Table 3).

### Candidate Genes and Associated Quantitative Trait Loci Under Selection

**FIGURE 4:**
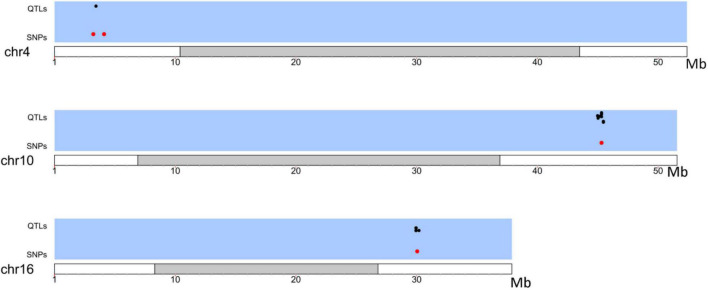
Distribution of QTLs (black) in LD with SNPs under selection (Red) between Brazilian and Asian Genotypes.

The upstream and downstream genomic regions within the LD decay distance (230 kb) of each SNP under selection were analyzed. Previously described QTLs (available at the Soybase’s QTL database) present in those regions are reported in Supplementary Table 4. Fourteen QTLs under selection were identified between the Brazilian and Asian genotypes (Figure 4 and Supplementary Table 4). We found QTLs associated with characteristics of relevance to the adaptation and commercial use of soybean in Brazil, for example, the number of days to flowering, number of days to maturity, plant height, and pod shattering.

SNP 1.5 is located inside the *E2* gene, a *GIGANTEA* (*GI*) homologue. *E2* has a large impact on the number of days to flowering and maturity in soybean, with few functional differences to its homologue in *Arabidopsis thaliana* (Watanabe et al., 2011; Wang Y. et al., 2016). The *GI* gene mediates the interaction between the circadian oscillator and *CONSTANS* (*CO*) to promote flowering. There is evidence of the conservation of this mechanism in several plant species, including soybean, thus making *E2* one of the main agents controlling flowering and maturity in this crop (Fowler et al., 1999; Suárez-López et al., 2001; Mizoguchi et al., 2005; Nogueira, 2011; Haider, 2014; Lin et al., 2020; Miranda et al., 2020).

**FIGURE 5:**
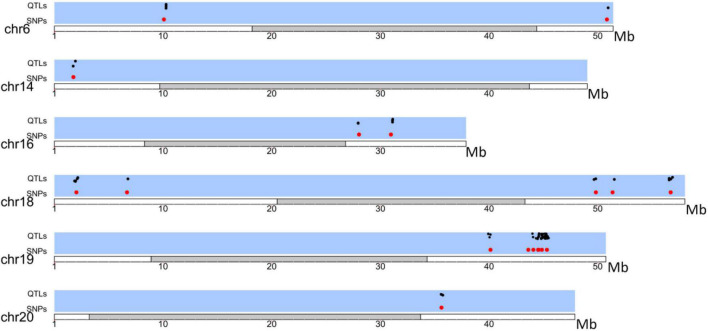
Distribution of QTLs (black) in LD with SNPs under selection (Red) among Brazilian companies.

Eighty-two QTLs under selection were identified among different Brazilian companies’ portfolios (Supplementary Table 5 and Figure 5). QTLs involved in the number of days to flowering and maturity; protein, oil, and lipid content; resistance to several pathogens; productivity components (seed weight, number of seeds per plant); and plant architecture (height, node number, stem shape, and determinacy) were identified within the genomic regions surrounding the SNPs under selection.

Several genes known to play important roles in the control of these phenotypes can also be found in the same genomic regions. Three genes controlling cycle duration in soybean can be found in the regions under selection. The gene *GmFT2a* is 141,137 bp away from SNP 2.12. *GmFT2a* is one of the 12 *FLOWERING LOCUS T* (*FT*) genes found in soybean and is the causal gene for the *E9* locus, which controls flowering in soybean (Kong et al., 2010, 2014; Li et al., 2014; Nan et al., 2014; Zhao et al., 2016; Wu et al., 2017; Cai et al., 2018; Lin et al., 2020). The *FT* gene is conserved in several plant species. In *A. thaliana*, it is the gene to which several signaling pathways converge to, integrating the photoperiod, temperature, vernalization, and light quality signaling pathways to regulate flowering (Amasino, 2010; Wickland and Hanzawa, 2015). In soybean, *GmFT2a* interacts with the *FDL19* transcription factor to stimulate the expression of *APETALA1* homologues, promoting flowering (Nan et al., 2014).

The SNP 2.19 under selection is located 197,494 bp from *Glyma.19g13800*, which is a homologue of *AT2G41710*, an endogenous gene in *A. thaliana* that belongs to the *APETALA 2* (*AP*) family. This gene family is directly involved in the control of flowering and seed development in *A. thaliana.* Various authors have already demonstrated that the roles of this gene family are conserved in several species, including soybean (Jofuku et al., 1994; Yant et al., 2010; Lei et al., 2019; Jiang et al., 2020).

SNPs 2.22, 2.23, 2.24, and 2.25 are located inside regions associated with cycle duration and several plant architecture components such as growth habit, plant height, and node number. Previous works that detected such QTLs in this region point to *Dt1* as a causal gene (Zhang et al., 2015; Contreras-Soto et al., 2017b; Mao et al., 2017). *Dt1* is a *TERMINAL FLOWER 1* (*TFL-1*) homologue; *TFL1* regulates growth habit and flowering in *A. thaliana* (Ratcliffe et al., 1998; Liu et al., 2010; Hanano and Goto, 2011; Goretti et al., 2020). Although some authors indicate that *Dt1* is involved in flowering regulation in soybean (Zhang et al., 2015; Mao et al., 2017), several studies have shown that this gene is sub-functionalized in soybean (Liu et al., 2010; Tian et al., 2010). However, a recent work from Yue et al. (2021) contradicts this notion by demonstrating that *Dt1* can interact with *GmFT5a* to control the number of days to flowering in soybean.

In chromosome 14, SNP 2.7 is inside a gene that codes for a toll-like interleukin receptor. This gene has been considered a causal gene of the QTL associated with *Diaporthe phaseolorum* resistance (Chang et al., 2016). In chromosome 18, SNP 2.13 is in a region that contains a QTL associated with *Fusarium virguliforme* and *Heterodera glycines* resistance (Wen et al., 2014; Chang et al., 2016). This QTL is a gene complex that includes *GmRLK18-1*, a leucine-rich repeat receptor-like protein kinase involved in resistance to sudden death syndrome (*F. virguliforme*) and the soybean cyst nematode (*H. glycines*) (Srour et al., 2012; Wen et al., 2014).

SNP 2.15 is 53,313 bp away from *Glyma.18g211100*, which codes for a peroxidase. Peroxidases play an important role in pathogen resistance, taking part in lignin, suberin, phytoalexin, and reactive oxygen species synthesis. These substances participate in the hypersensitivity response that causes controlled cell death to limit an area of infection and pathogen development (Almagro et al., 2009).

A gene associated with the number of seeds per pod is in one of the genomic regions under selection surrounding SNP 2.27. *Gm-JAG1* was identified by Fang et al. (2017) as the probable causal gene for *Seed-set1-g53.1. GM-JAG1*, positioned 206,523 bp from SNP 2.27, is a *JAG* homologue that in *A. thaliana* encodes a zinc finger-like protein that regulates organ growth and development (Ohno et al., 2004; Jeong et al., 2013; Sayama et al., 2017).

## Discussion

### Genotyping by Sequencing

Most of the polymorphic SNPs detected are concentrated in euchromatic regions. This is expected because the GBS technique is based on reducing genome complexity using methylation-sensitive restriction enzymes to focus sequencing to low-methylated regions, with higher gene concentration (Elshire et al., 2011; Sonah et al., 2013). Soybean is an autogamous plant, which explains the high level of homozygosity detected in the markers. This has been widely documented by other authors who have worked with soybean and other autogamous crops (Cockram et al., 2010; Mesquita, 2017; Sherman-Broyles et al., 2017; Li et al., 2020). The prevalence of SNPs classified as transitions corroborates values found in the literature (Van et al., 2005; Shu et al., 2011; dos Santos et al., 2016). Transversions more frequently change the translated amino acid sequence in comparison to transitions. This leads to transitions being under weaker purifying selection pressure, increasing their frequency in the genome relative to transversions (Guo et al., 2017).

### Population Structure and Genetic Diversity

The population structure analysis indicated the existence of three subpopulations originating from different geographic regions. The Asian genotypes were the most distinct group compared to the rest of the panel, with higher π-values as well as smaller linkage blocks, indicating higher genetic diversity than the Brazilian genotypes. These results corroborate previous authors’ findings that Asian genotypes have higher genetic diversity than cultivars from other regions (Li et al., 2008; Liu et al., 2017; Bruce et al., 2019). This high diversity is mainly because this is the region of domestication and where the center of origin of the crop is located (Xu et al., 2002; Guo et al., 2010; Li et al., 2010; Han et al., 2016; Wang J. et al., 2016; Jeong et al., 2019).

Average *F*_*ST*_ values among germplasms from different Brazilian breeding programs were markedly smaller than those between Asian and Brazilian genotypes, indicating low genetic divergence among Brazilian genotypes. This low genetic diversity compared to Asian genotypes corroborates previous works that have assessed genetic diversity in the Brazilian soybean (Priolli et al., 2013; Wysmierski and Vello, 2013; Gwinner et al., 2017). Founding events are strong diversity bottlenecks, and soybean went through several of these before being introduced into Brazil in the 1960s (Hiromoto and Vello, 1986; Hyten et al., 2006; Banks et al., 2013).

The distance at which LD decays is in accordance with previous works in soybean (Wen et al., 2014; Mao et al., 2017; Wang et al., 2018), with higher values observed for Brazilian genotypes. This was expected, given that these accessions went through rigorous artificial selection processes and more diversity bottlenecks in comparison to the Asian genotypes. The presence of larger linkage blocks in populations developed by modern breeding programs has been described by several authors (Hyten et al., 2007; Song et al., 2015; Wang J. et al., 2016). Brazilian germplasms have a narrow genetic base due to the low number of ancestors contributing to a large part of the allelic diversity present in soybean cultivars (Hiromoto and Vello, 1986; Priolli et al., 2013; Wysmierski and Vello, 2013). This small effective population size leads to the formation of large linkage blocks, resulting in a longer LD decay distance (Song et al., 2015).

Factors such as RMG and company of origin also had a significant influence on population stratification when the Brazilian genotypes were analyzed separately. We observed the formation of distinct groups of early maturing and late maturing cultivars. The influence of RMG on population structure has already been described (Žulj Mihaljević et al., 2020). Contreras-Soto et al. (2017a) also observed a link between RMG and population stratification in a Brazilian soybean panel. These authors also demonstrated the importance of the company of origin on population structure. The formation of a subpopulation comprised of late maturing cultivars was also observed by Gwinner et al. (2017) using a panel of 77 Brazilian soybean genotypes.

The RMG of a cultivar defines the latitude the genotype is adapted to Alliprandini et al. (2009), Cavassim et al. (2013), and Zdziarski et al. (2018). Thus, panel stratification between cultivars adapted to southern growing environments and cultivars better adapted to the environmental conditions at lower latitudes is expected. The growing environments in different latitudes differ not only in photoperiod length, but also in soil pH, nutrient availability, average temperature, and precipitation (Kaster and Farias, 2012; Lopes and Guimarães Guilherme, 2016). Therefore, breeding is directed differently depending on the latitude for which the program aims to develop cultivars, which could explain the stratification observed here.

We also observed that cultivars developed by the same company showed higher genetic similarity among themselves. The effect of company of origin on population stratification has previously been demonstrated in tropical soybean cultivars (Contreras-Soto et al., 2017a). Cultivars developed by the same company frequently originate from the same breeding program, and therefore, a lower level of genetic dissimilarity is expected.

When analyzed by company, the population structure revealed interesting patterns. A significant portion of the portfolios from companies such as TMG, Bayer, Corteva, and Embrapa belong to the same subpopulation as PI559369 (Lee 68), indicating high genetic similarity. Lee 68 originated from the backcrossing of Lee × Arksoy into Lee. Lee, in turn, is a progeny from the cross between S-100 and C.N.S, the two main ancestors responsible for the genetic base of Brazilian soybeans (Wysmierski and Vello, 2013).

Genotypes from companies such as GDM, Nidera, and Syngenta are notably similar in genetic constitution to North American lines from crosses involving Williams. Although Williams isn’t among the main ancestors contributing to the Brazilian genetic base, it is possibly through Williams that relevant ancestors such as Dunfield, Mandarin, Manchu, Peking, Richland, and Mukden contribute the base (Wysmierski, 2010; Priolli et al., 2013). These cultivars also tend to present with a shorter cycle duration and adaptation to higher latitudes.

### Selection Signatures in the Soybean Genome

A Bayesian approach was adopted through BayeScan software to detect genomic regions under selection between Brazilian and Asian genotypes, North American and Brazilian genotypes, and among Brazilian genotypes from different breeding programs. No SNPs under selection were detected between the North American and Brazilian genotypes. Seven SNPs under selection distributed in six genomic regions were detected in the Brazilian × Asian genotypes test. Genomic regions under selection between populations from different geographic origins are often associated with phenotypes controlling the species adaptability to different growing environments (Günther and Coop, 2013; Li et al., 2020; Saleem et al., 2021).

That six of the seven SNPs under selection were monomorphic in the Brazilian subpopulation suggests that these markers are associated with characteristics of relevance to the crop’s adaptability to Brazilian growing environments, although the possibility that random genetic drift is causing the fixation of these markers cannot be ignored. However, the SNPs under selection are not randomly distributed throughout the genome, but instead are located close to QTLs and genes specifically associated with phenotypes of great relevance to regional adaptation such as the number of days to flowering and maturity. This is strong evidence that the fixation in these loci wasn’t caused by random genetic drift (Hartl and Clark, 2007).

A larger set of SNPs under selection was detected among the Brazilian breeding programs, probably due to a higher number of groups being included in the analysis. Outlier SNPs are in LD with the QTLs associated with phenotypes of great relevance to breeders such as cycle duration, pathogen resistance, water use efficiency, and tolerance to nutrient deficiency. Brazil is a country where soybean growing environments span several latitudes, which demands from the crop the ability to adapt to environments with variable photoperiods, pathogen incidence, fertility, and precipitation. Studying the genomic regions under selection among breeding programs allows us to identify the QTLs relevant to regional adaptation (Jun et al., 2011).

It’s important to note that genomic regions of interest to breeders are constantly under severe selection pressure, leading to a reduction of diversity in those regions, which could culminate with their fixation. This reduction in diversity can be detrimental to the breeding programs’ ability to continually develop highly productive and adaptable cultivars (Hyten et al., 2006). There is a notable lack of studies that have looked at selection signatures in Brazilian soybean cultivars. Here, we identified 34 SNPs under selection which are in LD with 96 QTLs; many of these are of agronomic importance to soybean’s productivity and adaptation in Brazil. The genomic regions we identified can be explored by breeders aiming to increase the useful genetic diversity in Brazilian soybean germplasms and to develop cultivars able to adapt to the many Brazilian growing environments.

## Data Availability Statement

The data used in the study has been uploaded to a repository and can be found here: https://doi.org/10.5281/zenodo.6362858.

## Author Contributions

HM: conceptualization, data curation, formal analysis, investigation, methodology, software, visualization, writing—original draft, and writing—review and editing. LP: conceptualization, supervision, validation, and writing—review and editing. AM, GS, and JM: conceptualization, supervision, validation, writing—review and editing, resources, project administration, and funding acquisition. All authors contributed to the article and approved the submitted version.

## Conflict of Interest

The authors declare that the research was conducted in the absence of any commercial or financial relationships that could be construed as a potential conflict of interest.

## Publisher’s Note

All claims expressed in this article are solely those of the authors and do not necessarily represent those of their affiliated organizations, or those of the publisher, the editors and the reviewers. Any product that may be evaluated in this article, or claim that may be made by its manufacturer, is not guaranteed or endorsed by the publisher.

## Footnotes

1
www.soybase.org

## Supplementary Material

Supplementary Figure 1 Cluster analysis using the UPGMA method on genetic distances based on IBS among 247 Brazilian soybean cultivars, branches are colored by RMG whereas Cultivar labels are colored based on company of origin.
