# Supplementary material for: Genetic Diversity and Selection Footprints in the Genome of Brazilian Soybean Cultivars
Source: Front Plant Sci. 2022 Mar 30;13:842571. doi: 10.3389/fpls.2022.842571 (PMC9006619; doi:10.3389/fpls.2022.842571)
Supplement: Supplementary file 1 [file Table_1.DOCX]

**Supplementary Table 1 – Soybean accessions composing the panel. Classified as genotypes developed by Breeding Programs or collected materials.**

| **Accession** | **Origin*** | **Company** | **Country** | **RMG** |
| --- | --- | --- | --- | --- |
| **5D6215 IPRO** | **BP** | **TMG** | **Brazil** | **6.4** |
| **5G7315 IPRO** | **BP** | **Nidera** | **Brazil** | **7.3** |
| **A7002** | **BP** | **Nidera** | **Brazil** | **7** |
| **A7321 RG** | **BP** | **Nidera** | **Brazil** | **7.1** |
| **ANsc 89109** | **BP** | **Agronorte** | **Brazil** | **8.9** |
| **AS3797 IPRO** | **BP** | **Bayer** | **Brazil** | **7.9** |
| **AS3820 IPRO** | **BP** | **Bayer** | **Brazil** | **8.2** |
| **BMX APOLO RR** | **BP** | **GDM** | **Brazil** | **5.5** |
| **BMX BONUS IPRO** | **BP** | **GDM** | **Brazil** | **7.9** |
| **BMX BRAVA RR** | **BP** | **GDM** | **Brazil** | **6.3** |
| **BMX CERTA IPRO** | **BP** | **GDM** | **Brazil** | **8** |
| **BMX CLASSE RR** | **BP** | **GDM** | **Brazil** | **7.6** |
| **BMX DELTA IPRO** | **BP** | **GDM** | **Brazil** | **5.9** |
| **BMX DESAFIO RR** | **BP** | **GDM** | **Brazil** | **7.4** |
| **BMX ELITE IPRO** | **BP** | **GDM** | **Brazil** | **5.5** |
| **BMX ENERGIA RR** | **BP** | **GDM** | **Brazil** | **5.3** |
| **BMX EXTRA IPRO** | **BP** | **GDM** | **Brazil** | **7.4** |
| **BMX EXTREMA IPRO** | **BP** | **GDM** | **Brazil** | **8.1** |
| **BMX FIBRA IPRO** | **BP** | **GDM** | **Brazil** | **6.4** |
| **BMX FLECHA IPRO** | **BP** | **GDM** | **Brazil** | **6.6** |
| **BMX FOCO IPRO** | **BP** | **GDM** | **Brazil** | **7.4** |
| **BMX FORÇA RR** | **BP** | **GDM** | **Brazil** | **6.2** |
| **BMX FURIA RR** | **BP** | **GDM** | **Brazil** | **7.3** |
| **BMX GARRA IPRO** | **BP** | **GDM** | **Brazil** | **6.3** |
| **BMX ICONE IPRO** | **BP** | **GDM** | **Brazil** | **6.8** |
| **BMX LANÇA IPRO** | **BP** | **GDM** | **Brazil** | **5.8** |
| **BMX MAGNA RR** | **BP** | **GDM** | **Brazil** | **6.2** |
| **BMX PONTA IPRO** | **BP** | **GDM** | **Brazil** | **6.6** |
| **BMX POTÊNCIA RR** | **BP** | **GDM** | **Brazil** | **6.7** |
| **BMX PRISMA IPRO** | **BP** | **GDM** | **Brazil** | **7.1** |
| **BMX RAIO IPRO** | **BP** | **GDM** | **Brazil** | **5** |
| **BMX TORNADO RR** | **BP** | **GDM** | **Brazil** | **6.2** |
| **BMX TURBO RR** | **BP** | **GDM** | **Brazil** | **5.8** |
| **BMX ULTRA IPRO** | **BP** | **GDM** | **Brazil** | **7.5** |
| **BMX ÚNICA IPRO** | **BP** | **GDM** | **Brazil** | **6.8** |
| **BMX VANGUARDA IPRO** | **BP** | **GDM** | **Brazil** | **6** |
| **BMX ZEUS IPRO** | **BP** | **GDM** | **Brazil** | **5.5** |
| **BR5.8 IPRO** | **BP** | **Embrapa** | **Brazil** | **-** |
| **BR5.9 IPRO** | **BP** | **Embrapa** | **Brazil** | **-** |
| **BRS1010 IPRO** | **BP** | **Embrapa** | **Brazil** | **6.1** |
| **BRS133** | **BP** | **Embrapa** | **Brazil** | **7.4** |
| **BRS134** | **BP** | **Embrapa** | **Brazil** | **-** |
| **BRS184** | **BP** | **Embrapa** | **Brazil** | **6.9** |
| **BRS206** | **BP** | **Embrapa** | **Brazil** | **6.8** |
| **BRS231** | **BP** | **Embrapa** | **Brazil** | **-** |
| **BRS232** | **BP** | **Embrapa** | **Brazil** | **6.9** |
| **BRS246 RR** | **BP** | **Embrapa** | **Brazil** | **7.2** |
| **BRS268** | **BP** | **Embrapa** | **Brazil** | **-** |
| **BRS388 RR** | **BP** | **Embrapa** | **Brazil** | **6.4** |
| **BRS517** | **BP** | **Embrapa** | **Brazil** | **7.1** |
| **BRS519** | **BP** | **Embrapa** | **Brazil** | **7.1** |
| **BRS533** | **BP** | **Embrapa** | **Brazil** | **6.9** |
| **BRS5601 RR** | **BP** | **Embrapa** | **Brazil** | **5.6** |
| **BRS6203 RR** | **BP** | **Embrapa** | **Brazil** | **6.2** |
| **BRS6980** | **BP** | **Embrapa** | **Brazil** | **6.9** |
| **BRS7380 RR** | **BP** | **Embrapa** | **Brazil** | **7.3** |
| **BRS7980** | **BP** | **Embrapa** | **Brazil** | **7.9** |
| **BRS8990 IPRO** | **BP** | **Embrapa** | **Brazil** | **8.9** |
| **BRSValiosa RR** | **BP** | **Embrapa** | **Brazil** | **8.1** |
| **BS2601 RR** | **BP** | **BASF** | **Brazil** | **6.1** |
| **C2375 IPRO** | **BP** | **TMG** | **Brazil** | **7.5** |
| **C2379 IPRO** | **BP** | **TMG** | **Brazil** | **7.9** |
| **C7370 IPRO** | **BP** | **TMG** | **Brazil** | **7** |
| **CD202 IPRO** | **BP** | **Corteva** | **Brazil** | **6.4** |
| **CD208** | **BP** | **Corteva** | **Brazil** | **6.9** |
| **CD217** | **BP** | **Corteva** | **Brazil** | **8** |
| **CD2590 IPRO** | **BP** | **Corteva** | **Brazil** | **5.9** |
| **CD2610 IPRO** | **BP** | **Corteva** | **Brazil** | **6.1** |
| **CD2620 IPRO** | **BP** | **Corteva** | **Brazil** | **6.2** |
| **CD2630 RR** | **BP** | **Corteva** | **Brazil** | **6.3** |
| **CD2694 IPRO** | **BP** | **Corteva** | **Brazil** | **6.9** |
| **CD2700 IPRO** | **BP** | **Corteva** | **Brazil** | **7** |
| **CD2720 IPRO** | **BP** | **Corteva** | **Brazil** | **7.2** |
| **CD2750 IPRO** | **BP** | **Corteva** | **Brazil** | **7.5** |
| **CG7262 RR** | **BP** | **Caraíba** | **Brazil** | **7.2** |
| **CG7665 RR** | **BP** | **Caraíba** | **Brazil** | **7.6** |
| **CG8166 RR** | **BP** | **Caraíba** | **Brazil** | **8.1** |
| **CZ36B31 IPRO** | **BP** | **BASF** | **Brazil** | **6.3** |
| **CZ48B32 IPRO** | **BP** | **BASF** | **Brazil** | **8.3** |
| **DM5958RSF** | **BP** | **GDM** | **Brazil** | **5.8** |
| **DM66I68 IPRO** | **BP** | **GDM** | **Brazil** | **6.6** |
| **DM66I68RSF IPRO** | **BP** | **GDM** | **Brazil** | **6.6** |
| **DM68I69 IPRO** | **BP** | **GDM** | **Brazil** | **6.8** |
| **DM68I69RSF IPRO** | **BP** | **GDM** | **Brazil** | **6.8** |
| **DM75I76 IPRO** | **BP** | **GDM** | **Brazil** | **7.5** |
| **DM75I76RSF IPRO** | **BP** | **GDM** | **Brazil** | **7.5** |
| **DM80I79RSF IPRO** | **BP** | **GDM** | **Brazil** | **8** |
| **DM81I84 IPRO** | **BP** | **GDM** | **Brazil** | **8.1** |
| **DS6716 IPRO** | **BP** | **TMG** | **Brazil** | **6.7** |
| **FPS1755 IPRO** | **BP** | **GDM** | **Brazil** | **5.5** |
| **FPS Antares RR** | **BP** | **GDM** | **Brazil** | **6.8** |
| **FPS Solar IPRO** | **BP** | **GDM** | **Brazil** | **6.3** |
| **FPS Urano RR** | **BP** | **GDM** | **Brazil** | **6.2** |
| **FTR2155 RR** | **BP** | **FTS** | **Brazil** | **5.8** |
| **FTR2161 RR** | **BP** | **FTS** | **Brazil** | **6.1** |
| **FTR3156 IPRO** | **BP** | **FTS** | **Brazil** | **5.6** |
| **FTR4149 IPRO** | **BP** | **FTS** | **Brazil** | **4.9** |
| **FTR4153 IPRO** | **BP** | **FTS** | **Brazil** | **5.3** |
| **FTR4160 IPRO** | **BP** | **FTS** | **Brazil** | **6** |
| **FTS Master RR** | **BP** | **FTS** | **Brazil** | **8.2** |
| **GB874 RR** | **BP** | **Bayer** | **Brazil** | **8.7** |
| **GNZ550S RR** | **BP** | **Limagrain** | **Brazil** | **5.5** |
| **HO JURUENA IPRO** | **BP** | **GDM** | **Brazil** | **8.7** |
| **HO MARACAÍ IPRO** | **BP** | **GDM** | **Brazil** | **7.7** |
| **HO PARANAÍBA IPRO** | **BP** | **GDM** | **Brazil** | **7.4** |
| **M5410 IPRO** | **BP** | **Bayer** | **Brazil** | **5.4** |
| **M5838 IPRO** | **BP** | **Bayer** | **Brazil** | **5.8** |
| **M5892 IPRO** | **BP** | **Bayer** | **Brazil** | **5.8** |
| **M5917 IPRO** | **BP** | **Bayer** | **Brazil** | **5.9** |
| **M6952 IPRO** | **BP** | **Bayer** | **Brazil** | **6.9** |
| **M6972 IPRO** | **BP** | **Bayer** | **Brazil** | **6.9** |
| **M7110 IPRO** | **BP** | **Bayer** | **Brazil** | **7.1** |
| **M7198 IPRO** | **BP** | **Bayer** | **Brazil** | **7.1** |
| **M7739 IPRO** | **BP** | **Bayer** | **Brazil** | **7.7** |
| **M7901** | **BP** | **Bayer** | **Brazil** | **-** |
| **M8349 IPRO** | **BP** | **Bayer** | **Brazil** | **8.3** |
| **M8372 IPRO** | **BP** | **Bayer** | **Brazil** | **8.3** |
| **M8644 IPRO** | **BP** | **Bayer** | **Brazil** | **8.6** |
| **M8766 RR** | **BP** | **Bayer** | **Brazil** | **8.7** |
| **NA5909** | **BP** | **Nidera** | **Brazil** | **5.9** |
| **NA5947 IPRO** | **BP** | **Nidera** | **Brazil** | **-** |
| **NA7337 RR** | **BP** | **Nidera** | **Brazil** | **7.5** |
| **NK3363** | **BP** | **Syngenta** | **Brazil** | **6.3** |
| **NS4823 RR** | **BP** | **Nidera** | **Brazil** | **4.8** |
| **NS4901 RR** | **BP** | **Nidera** | **Brazil** | **4.9** |
| **NS5160 IPRO** | **BP** | **Nidera** | **Brazil** | **5.1** |
| **NS5258 RR** | **BP** | **Nidera** | **Brazil** | **5.2** |
| **NS5290 RR** | **BP** | **Nidera** | **Brazil** | **5.2** |
| **NS5445 IPRO** | **BP** | **Nidera** | **Brazil** | **5.4** |
| **NS5727 IPRO** | **BP** | **Nidera** | **Brazil** | **5.7** |
| **NS5858 RR** | **BP** | **Nidera** | **Brazil** | **5.8** |
| **NS5959 IPRO** | **BP** | **Nidera** | **Brazil** | **5.9** |
| **NS6006 IPRO** | **BP** | **Nidera** | **Brazil** | **6** |
| **NS6209 RR** | **BP** | **Nidera** | **Brazil** | **6.2** |
| **NS6262 RR** | **BP** | **Nidera** | **Brazil** | **6.2** |
| **NS6601 IPRO** | **BP** | **Nidera** | **Brazil** | **6.6** |
| **NS6700 IPRO** | **BP** | **Nidera** | **Brazil** | **6.7** |
| **NS6906 IPRO** | **BP** | **Nidera** | **Brazil** | **7** |
| **NS6909 IPRO** | **BP** | **Nidera** | **Brazil** | **6.9** |
| **NS7000 IPRO** | **BP** | **Nidera** | **Brazil** | **7** |
| **NS7007 IPRO** | **BP** | **Nidera** | **Brazil** | **7.1** |
| **NS7011 IPRO** | **BP** | **Nidera** | **Brazil** | **7** |
| **NS7100 RR** | **BP** | **Nidera** | **Brazil** | **7.1** |
| **NS7202 IPRO** | **BP** | **Nidera** | **Brazil** | **7.2** |
| **NS7209 IPRO** | **BP** | **Nidera** | **Brazil** | **7.2** |
| **NS7237 IPRO** | **BP** | **Nidera** | **Brazil** | **7.2** |
| **NS7300 IPRO** | **BP** | **Nidera** | **Brazil** | **7.3** |
| **NS7338 IPRO** | **BP** | **Nidera** | **Brazil** | **7.3** |
| **NS7447 IPRO** | **BP** | **Nidera** | **Brazil** | **7.4** |
| **NS7505 IPRO** | **BP** | **Nidera** | **Brazil** | **7.5** |
| **NS7667 IPRO** | **BP** | **Nidera** | **Brazil** | **7.6** |
| **NS7670 RR** | **BP** | **Nidera** | **Brazil** | **7.6** |
| **NS7709 IPRO** | **BP** | **Nidera** | **Brazil** | **7.2** |
| **NS7780 IPRO** | **BP** | **Nidera** | **Brazil** | **7.2** |
| **NS7901 RR** | **BP** | **Nidera** | **Brazil** | **7.9** |
| **NS8338 IPRO** | **BP** | **Nidera** | **Brazil** | **8.3** |
| **NS8383 RR** | **BP** | **Nidera** | **Brazil** | **8.3** |
| **NS8399 IPRO** | **BP** | **Nidera** | **Brazil** | **8.3** |
| **P95R51 RR** | **BP** | **Corteva** | **Brazil** | **5.5** |
| **P95Y52 RR** | **BP** | **Corteva** | **Brazil** | **5.5** |
| **P98C81** | **BP** | **Corteva** | **Brazil** | **8.8** |
| **P98Y11 RR** | **BP** | **Corteva** | **Brazil** | **8.1** |
| **P98Y12 RR** | **BP** | **Corteva** | **Brazil** | **8.1** |
| **PI103091** | **Collected** | **-** | **China** | **-** |
| **PI238109** | **Collected** | **-** | **Japan** | **-** |
| **PI274454** | **Collected** | **-** | **Japan** | **-** |
| **PI274456** | **Collected** | **-** | **Japan** | **-** |
| **PI291327** | **Collected** | **-** | **China** | **-** |
| **PI304218** | **Collected** | **-** | **China** | **-** |
| **PI306704A** | **Collected** | **-** | **Tanzania** | **-** |
| **PI307889B** | **Collected** | **-** | **India** | **-** |
| **PI340034** | **Collected** | **-** | **South Korea** | **-** |
| **PI340049** | **Collected** | **-** | **South Korea** | **-** |
| **PI378682A** | **Collected** | **-** | **Japan** | **-** |
| **PI393538** | **Collected** | **-** | **Japan** | **-** |
| **PI398775** | **Collected** | **-** | **South Korea** | **-** |
| **PI399017** | **Collected** | **-** | **South Korea** | **-** |
| **PI399073** | **Collected** | **-** | **South Korea** | **-** |
| **PI399079** | **Collected** | **-** | **South Korea** | **-** |
| **PI399080** | **Collected** | **-** | **South Korea** | **-** |
| **PI428692** | **Collected** | **-** | **India** | **-** |
| **PI518671** | **BP** | **-** | **USA** | **-** |
| **PI547677** | **BP** | **-** | **USA** | **-** |
| **PI547764** | **BP** | **-** | **USA** | **-** |
| **PI547788** | **BP** | **-** | **USA** | **-** |
| **PI547791** | **BP** | **-** | **USA** | **-** |
| **PI547794** | **BP** | **-** | **USA** | **-** |
| **PI547834** | **BP** | **-** | **USA** | **-** |
| **PI547838** | **BP** | **-** | **USA** | **-** |
| **PI547841** | **BP** | **-** | **USA** | **-** |
| **PI547842** | **BP** | **-** | **USA** | **-** |
| **PI547856** | **BP** | **-** | **USA** | **-** |
| **PI547862** | **BP** | **-** | **USA** | **-** |
| **PI547874** | **BP** | **-** | **USA** | **-** |
| **PI547876** | **BP** | **-** | **USA** | **-** |
| **PI548571** | **BP** | **-** | **Canada** | **-** |
| **PI548573** | **BP** | **-** | **Canada** | **-** |
| **PI548622** | **BP** | **-** | **USA** | **-** |
| **PI556782** | **BP** | **-** | **USA** | **-** |
| **PI556912** | **BP** | **-** | **USA** | **-** |
| **PI559369** | **BP** | **-** | **USA** | **-** |
| **PI567070A** | **Collected** | **-** | **Indonesia** | **-** |
| **PI567078** | **Collected** | **-** | **Indonesia** | **-** |
| **PI567088A** | **Collected** | **-** | **Indonesia** | **-** |
| **PI567132A** | **Collected** | **-** | **Indonesia** | **-** |
| **PI587585A** | **Collected** | **-** | **China** | **-** |
| **PI587598A** | **Collected** | **-** | **China** | **-** |
| **PI587600C** | **Collected** | **-** | **China** | **-** |
| **PI587604A** | **Collected** | **-** | **China** | **-** |
| **PI587645** | **Collected** | **-** | **China** | **-** |
| **PI587646** | **Collected** | **-** | **China** | **-** |
| **PI587715** | **Collected** | **-** | **China** | **-** |
| **PI587716C** | **Collected** | **-** | **China** | **-** |
| **PI587718** | **Collected** | **-** | **China** | **-** |
| **PI587728** | **Collected** | **-** | **China** | **-** |
| **PI587788A** | **Collected** | **-** | **China** | **-** |
| **PI587805** | **Collected** | **-** | **China** | **-** |
| **PI587809A** | **Collected** | **-** | **China** | **-** |
| **PI587819** | **Collected** | **-** | **China** | **-** |
| **PI587820A** | **Collected** | **-** | **China** | **-** |
| **PI587846A** | **Collected** | **-** | **China** | **-** |
| **PI587848** | **Collected** | **-** | **China** | **-** |
| **PI587860** | **Collected** | **-** | **China** | **-** |
| **PI587892A** | **Collected** | **-** | **China** | **-** |
| **PI587968A** | **Collected** | **-** | **China** | **-** |
| **PI587970A** | **Collected** | **-** | **China** | **-** |
| **PI587973A** | **Collected** | **-** | **China** | **-** |
| **PI587998G** | **Collected** | **-** | **China** | **-** |
| **PI588005A** | **Collected** | **-** | **China** | **-** |
| **PI588007A** | **Collected** | **-** | **China** | **-** |
| **PI588014A** | **Collected** | **-** | **China** | **-** |
| **PI588015D** | **Collected** | **-** | **China** | **-** |
| **PI588024B** | **Collected** | **-** | **China** | **-** |
| **PI588027C** | **Collected** | **-** | **China** | **-** |
| **PI588051** | **Collected** | **-** | **China** | **-** |
| **PI588053A** | **Collected** | **-** | **China** | **-** |
| **PI591505** | **BP** | **-** | **USA** | **-** |
| **PI591507** | **BP** | **-** | **USA** | **-** |
| **PI591509** | **BP** | **-** | **USA** | **-** |
| **PI591510** | **BP** | **-** | **USA** | **-** |
| **PI591511** | **BP** | **-** | **USA** | **-** |
| **PI591512** | **BP** | **-** | **USA** | **-** |
| **PI628850** | **BP** | **IAC** | **Brazil** | **-** |
| **PI632667** | **Collected** | **-** | **Vietnam** | **-** |
| **PI675661** | **BP** | **Embrapa** | **Brazil** | **9.2** |
| **PIQUIRI IPRO** | **BP** | **GDM** | **Brazil** | **7.9** |
| **PP8201 IPRO** | **BP** | **GDM** | **Brazil** | **7.4** |
| **Produza IPRO** | **BP** | **TMG** | **Brazil** | **6** |
| **ST797 IPRO** | **BP** | **BASF** | **Brazil** | **7.9** |
| **ST920 RR** | **BP** | **BASF** | **Brazil** | **9.2** |
| **SYN1157 RR** | **BP** | **Syngenta** | **Brazil** | **5.7** |
| **SYN1158 RR** | **BP** | **Syngenta** | **Brazil** | **5.8** |
| **SYN1163 RR** | **BP** | **Syngenta** | **Brazil** | **6.3** |
| **SYN1257 RR** | **BP** | **Syngenta** | **Brazil** | **5.7** |
| **SYN1258 RR** | **BP** | **Syngenta** | **Brazil** | **5.8** |
| **SYN1263 RR** | **BP** | **Syngenta** | **Brazil** | **6.3** |
| **SYN1283 RR** | **BP** | **Syngenta** | **Brazil** | **8.3** |
| **SYN13561 IPRO** | **BP** | **Syngenta** | **Brazil** | **5.8** |
| **SYN1359S IPRO** | **BP** | **Syngenta** | **Brazil** | **5.9** |
| **SYN13610 IPRO** | **BP** | **Syngenta** | **Brazil** | **6.3** |
| **SYN1363 RR** | **BP** | **Syngenta** | **Brazil** | **6.3** |
| **SYN1365 RR** | **BP** | **Syngenta** | **Brazil** | **6.5** |
| **SYN13670 IPRO** | **BP** | **Syngenta** | **Brazil** | **7.5** |
| **SYN13671 IPRO** | **BP** | **Syngenta** | **Brazil** | **7.3** |
| **SYN1561 IPRO** | **BP** | **Syngenta** | **Brazil** | **6.1** |
| **SYN1562 IPRO** | **BP** | **Syngenta** | **Brazil** | **6.2** |
| **SYN1687 IPRO** | **BP** | **Syngenta** | **Brazil** | **8.7** |
| **SYN3358 RR** | **BP** | **Syngenta** | **Brazil** | **5.8** |
| **TMG103 RR** | **BP** | **TMG** | **Brazil** | **8.3** |
| **TMG106 RR** | **BP** | **TMG** | **Brazil** | **8.3** |
| **TMG1066 RR** | **BP** | **TMG** | **Brazil** | **6.6** |
| **TMG1067 RR** | **BP** | **TMG** | **Brazil** | **6.7** |
| **TMG108 RR** | **BP** | **TMG** | **Brazil** | **8.9** |
| **TMG1161 RR** | **BP** | **TMG** | **Brazil** | **-** |
| **TMG1168 RR** | **BP** | **TMG** | **Brazil** | **6.8** |
| **TMG117 RR** | **BP** | **TMG** | **Brazil** | **8.5** |
| **TMG1174 RR** | **BP** | **TMG** | **Brazil** | **7.4** |
| **TMG1175 RR** | **BP** | **TMG** | **Brazil** | **7.5** |
| **TMG1176 RR** | **BP** | **TMG** | **Brazil** | **7.6** |
| **TMG1179 RR** | **BP** | **TMG** | **Brazil** | **7.9** |
| **TMG1180 RR** | **BP** | **TMG** | **Brazil** | **8** |
| **TMG1181 RR** | **BP** | **TMG** | **Brazil** | **8.1** |
| **TMG1182 RR** | **BP** | **TMG** | **Brazil** | **8.2** |
| **TMG1187 RR** | **BP** | **TMG** | **Brazil** | **8.7** |
| **TMG1188 RR** | **BP** | **TMG** | **Brazil** | **8.8** |
| **TMG123 RR** | **BP** | **TMG** | **Brazil** | **7.4** |
| **TMG125 RR** | **BP** | **TMG** | **Brazil** | **7.1** |
| **TMG1264 RR** | **BP** | **TMG** | **Brazil** | **6.4** |
| **TMG1266 RR** | **BP** | **TMG** | **Brazil** | **6.6** |
| **TMG127 RR** | **BP** | **TMG** | **Brazil** | **7.2** |
| **TMG1288 RR** | **BP** | **TMG** | **Brazil** | **8.8** |
| **TMG131 RR** | **BP** | **TMG** | **Brazil** | **8.3** |
| **TMG132 RR** | **BP** | **TMG** | **Brazil** | **8.5** |
| **TMG133 RR** | **BP** | **TMG** | **Brazil** | **8.5** |
| **TMG1759 RR** | **BP** | **TMG** | **Brazil** | **5.9** |
| **TMG2158 IPRO** | **BP** | **TMG** | **Brazil** | **5.8** |
| **TMG2165 IPRO** | **BP** | **TMG** | **Brazil** | **6.5** |
| **TMG2173 IPRO** | **BP** | **TMG** | **Brazil** | **7.3** |
| **TMG2179 IPRO** | **BP** | **TMG** | **Brazil** | **7.9** |
| **TMG2181 IPRO** | **BP** | **TMG** | **Brazil** | **8.1** |
| **TMG2182 IPRO** | **BP** | **TMG** | **Brazil** | **8.2** |
| **TMG2183 IPRO** | **BP** | **TMG** | **Brazil** | **8.3** |
| **TMG2185 IPRO** | **BP** | **TMG** | **Brazil** | **8.5** |
| **TMG2187 IPRO** | **BP** | **TMG** | **Brazil** | **8.7** |
| **TMG2281 IPRO** | **BP** | **TMG** | **Brazil** | **8.1** |
| **TMG2286 IPRO** | **BP** | **TMG** | **Brazil** | **8.6** |
| **TMG2364 IPRO** | **BP** | **TMG** | **Brazil** | **6.4** |
| **TMG2375 IPRO** | **BP** | **TMG** | **Brazil** | **7.5** |
| **TMG2378 IPRO** | **BP** | **TMG** | **Brazil** | **7.8** |
| **TMG2379 IPRO** | **BP** | **TMG** | **Brazil** | **7.9** |
| **TMG2381 IPRO** | **BP** | **TMG** | **Brazil** | **8.1** |
| **TMG2383 IPRO** | **BP** | **TMG** | **Brazil** | **8.3** |
| **TMG3001** | **BP** | **TMG** | **Brazil** | **-** |
| **TMG3002** | **BP** | **TMG** | **Brazil** | **-** |
| **TMG3003** | **BP** | **TMG** | **Brazil** | **-** |
| **TMG4001 RR** | **BP** | **TMG** | **Brazil** | **6.9** |
| **TMG4181** | **BP** | **TMG** | **Brazil** | **8.1** |
| **TMG4182** | **BP** | **TMG** | **Brazil** | **8.2** |
| **TMG4185** | **BP** | **TMG** | **Brazil** | **8.5** |
| **TMG4186** | **BP** | **TMG** | **Brazil** | **8.6** |
| **TMG4190** | **BP** | **TMG** | **Brazil** | **9** |
| **TMG5400 IPRO** | **BP** | **TMG** | **Brazil** | **6** |
| **TMG6228** | **BP** | **TMG** | **Brazil** | **-** |
| **TMG6400 IPRO** | **BP** | **TMG** | **Brazil** | **-** |
| **TMG7058 IPRO** | **BP** | **TMG** | **Brazil** | **5.8** |
| **TMG7059 IPRO** | **BP** | **TMG** | **Brazil** | **5.9** |
| **TMG7060 IPRO** | **BP** | **TMG** | **Brazil** | **6** |
| **TMG7061 IPRO** | **BP** | **TMG** | **Brazil** | **6.1** |
| **TMG7062 IPRO** | **BP** | **TMG** | **Brazil** | **6.2** |
| **TMG7063 IPRO** | **BP** | **TMG** | **Brazil** | **6.3** |
| **TMG7067 IPRO** | **BP** | **TMG** | **Brazil** | **6.7** |
| **TMG7161 RR** | **BP** | **TMG** | **Brazil** | **5.9** |
| **TMG7188 RR** | **BP** | **TMG** | **Brazil** | **8.8** |
| **TMG7260 IPRO** | **BP** | **TMG** | **Brazil** | **6** |
| **TMG7262 RR** | **BP** | **TMG** | **Brazil** | **6.2** |
| **TMG7363 RR** | **BP** | **TMG** | **Brazil** | **6.3** |
| **TMG7368 IPRO** | **BP** | **TMG** | **Brazil** | **6.7** |
| **TMG801** | **BP** | **TMG** | **Brazil** | **8.2** |
| **TMG803** | **BP** | **TMG** | **Brazil** | **8.7** |
| **TMG8551 RR** | **BP** | **TMG** | **Brazil** | **8.5** |
| **TMG CARAVANA RR** | **BP** | **TMG** | **Brazil** | **-** |
| **TMGRTRNCS** | **BP** | **TMG** | **Brazil** | **-** |
| **VTOP RR** | **BP** | **Syngenta** | **Brazil** | **6** |

***** BP – Breeding Program
