# Supplementary material for: Genetic Diversity and Selection Footprints in the Genome of Brazilian Soybean Cultivars
Source: Front Plant Sci. 2022 Mar 30;13:842571. doi: 10.3389/fpls.2022.842571 (PMC9006619; doi:10.3389/fpls.2022.842571)
Supplement: Supplementary file 2 [file Table_2.DOCX]

**Supplementary Table 2** – Outlier SNPs between Brazilian and Asian genotypes

| **SNP** | **Chromosome** | **Position (Mb)** | $\boldsymbol{-}\mathbf{log}_{\boldsymbol{10}} \boldsymbol{(q)}$ | | **alpha** |
| --- | --- | --- | --- | --- | --- |
| 1.1 | 4 | 3.215 | | 2 | 1.431 |
| 1.2 | 4 | 4.106 | | 1.85 | 1.535 |
| 1.3 | 4 | 4.106 | | 1.7 | 1.486 |
| 1.4 | 8 | 46.098 | | 1.004 | 0.942 |
| 1.5 | 10 | 45.31 | | 1.15 | 1.210 |
| 1.6 | 16 | 30.046 | | 1.29 | 1.214 |
| 1.7 | 19 | 17.204 | | 1.76 | 0.89 |
