# Supplementary material for: Genetic Diversity and Selection Footprints in the Genome of Brazilian Soybean Cultivars
Source: Front Plant Sci. 2022 Mar 30;13:842571. doi: 10.3389/fpls.2022.842571 (PMC9006619; doi:10.3389/fpls.2022.842571)
Supplement: Supplementary file 3 [file Table_3.DOCX]

**Supplementary Table 3** – Outlier SNPs among Brazilian breeding programs

| **SNP** | **Chromosome** | **Position (Mb)** | $\boldsymbol{-}\mathbf{log}_{\boldsymbol{10}} \boldsymbol{(q)}$ | | **alpha** |
| --- | --- | --- | --- | --- | --- |
| 2.1 | 6 | 10.081 | | 1.03 | -1.296 |
| 2.2 | 6 | 41.698 | | 1.31 | 1.026 |
| 2.3 | 6 | 50.843 | | 1.01 | 0.870 |
| 2.4 | 8 | 15.79 | | 1.08 | 1.071 |
| 2.5 | 12 | 7.504 | | 1.37 | 0.972 |
| 2.6 | 13 | 39.213 | | 1.75 | 0.954 |
| 2.7 | 14 | 1.751 | | 1.86 | 1.298 |
| 2.8 | 16 | 23.996 | | 2.31 | 1.435 |
| 2.9 | 16 | 25.073 | | 1.65 | 1.196 |
| 2.10 | 16 | 26.987 | | 1.21 | 1.068 |
| 2.11 | 16 | 28.034 | | 1.26 | 0.9 |
| 2.12 | 16 | 30.969 | | 3.52 | 1.442 |
| 2.13 | 18 | 2.027 | | 1.06 | 0.789 |
| 2.14 | 18 | 6.684 | | 1.41 | 1.149 |
| 2.15 | 18 | 49.826 | | 1.57 | 1.145 |
| 2.16 | 18 | 51.363 | | 1.45 | 1.291 |
| 2.17 | 18 | 56.707 | | 3.52 | 1.368 |
| 2.18 | 18 | 56.71 | | 2.14 | 1.162 |
| 2.19 | 19 | 40.132 | | 1.34 | 0.952 |
| 2.20 | 19 | 43.609 | | 1.17 | 0.887 |
| 2.21 | 19 | 44.075 | | 2.59 | 1.258 |
| 2.22 | 19 | 44.508 | | 2.43 | 1.214 |
| 2.23 | 19 | 44.576 | | 2.74 | 1.242 |
| 2.24 | 19 | 44.863 | | 1.11 | 0.788 |
| 2.25 | 19 | 45.322 | | 1.50 | 0.918 |
| 2.26 | 20 | 35.621 | | 1.14 | 0.805 |
| 2.27 | 20 | 35.621 | | 2.05 | 1.076 |
