# Supplementary material for: Genetic Diversity and Selection Footprints in the Genome of Brazilian Soybean Cultivars
Source: Front Plant Sci. 2022 Mar 30;13:842571. doi: 10.3389/fpls.2022.842571 (PMC9006619; doi:10.3389/fpls.2022.842571)
Supplement: Supplementary file 4 [file Table_4.DOCX]

**Supplementary Table 4**– QTL in LD with outlier SNPs between Brazilian and Asian Genotypes described on Soybase.

| **SNP** | **Position (Mb)** | **Chromosome** | **Associated QTL*** |
| --- | --- | --- | --- |
| 1.1 | 3.215 | 4 | Seed isoflavone 1-g15 |
| 1.2; 1.3 | 4.106 – 4.106 | 4 | - |
| 1.4 | 46.098 | 8 | - |
| 1.5 | 45.311 | 10 | DTF 5-g1.2; DTF 5-g15.2; DTF 5-g1.3; DTF 5-g15.3; DTM5-g2.3; DTM5-g2.2; CAN 1-g6; Node number 1-g1 |
| 1.6 | 30.046 | 16 | PH 6-g18; PH 1-g18; Pod shattering 1-g1.2; Pod shattering 1-g1.3 |
| 1.7 | 17.204 | 19 | - |

**DTF*: days to flowering*; DTM*: Days to maturity; *CAN*: canopy cover; *PH*: plant height
