# Supplementary material for: Genetic Diversity and Selection Footprints in the Genome of Brazilian Soybean Cultivars
Source: Front Plant Sci. 2022 Mar 30;13:842571. doi: 10.3389/fpls.2022.842571 (PMC9006619; doi:10.3389/fpls.2022.842571)
Supplement: Supplementary file 5 [file Table_5.DOCX]

**Supplementary Table 5** – QTL in LD with outlier SNPs among Brazilian breeding companies

| **SNP** | **Chromosome** | **Position (Mb)** | **Associated QTL*** | |
| --- | --- | --- | --- | --- |
| 2.1 | 6 | 10.081 | | Seed Met 1-g1; DFTM 4-g7; DFTM 2-g7 |
| 2.2 | 6 | 41.698 | | - |
| 2.3 | 6 | 50.843 | | Seed set 1-g7 |
| 2.4 | 8 | 15.79 | | - |
| 2.5 | 12 | 7.504 | | - |
| 2.6 | 13 | 39.213 | | - |
| 2.7 | 14 | 1.751 | | Diaporthe stem canker 1-g1; Diaporthe stem canker 1-g2 |
| 2.8 | 16 | 23.996 | | - |
| 2.9 | 16 | 25.073 | | - |
| 2.10 | 16 | 26.987 | | - |
| 2.11 | 16 | 28.034 | | DTM5-g4.2; DTM5-g4.3 |
| 2.12 | 16 | 30.969 | | DTF 4-g64; DTF 4-g65 |
| 2.13 | 18 | 2.027 | | SDS 1-g32; SDS 1-g50; Velvetbean caterpillar 1-g1; Iron deficiency chlorosis 3-g12; SCN 1-g20 |
| 2.14 | 18 | 6.684 | | PROT 7-g27 |
| 2.15 | 18 | 49.826 | | SCN 2-g8; Sclero 3-g36 |
| 2.16 | 18 | 51.363 | | WUE 3-g30 |
| 2.17; 2.18 | 18 | 56.707 – 56.71 | | DTF 7-g23; DTF 2-g23; Seed isoflavone 1-g39; PH 3-g14 |
| 2.19 | 19 | 40.132 | | DTF 4-g76; PH 3-g15; PubDen 1-g17; SW 9-g5.1 |
| 2.20; 2.21 | 19 | 43.609 – 44.075 | | Node number 1-g2.1; Stem termination type 1-g1.1 |
| 2.22; 2.23; 2.24 | 19 | 44.508 – 44.863 | | PH 5-g3.1; Pod number 1-g2.1; Seed set 1-g55.1; Seed set 1-g50.1; Seed set 1-g55.2; PH 6-g24; PH 1-g24; AMIN 1-g25.1; Internode length 1-g2.1;Stem shape, main 1-g2.1; Internode length 1-g2.2; Lodging 1-g1.1; Seed set 1-g27.1; AMIN 1-g25.2; AMIN 1-g25.3; Seed set 1-g24.1; Seed set 1-g24.2; Seed set 1-g24.3; PH 3-g9 |
| 2.25 | 19 | 45.322 | | Lodging 1-g1.2; LeafArea 1-g3.1; LeafWidth 1-g5.1; LeafWidth 1-g5.2; Stem termination type 1-g1.2; Stem shape, main 1-g2.2; LeafArea 1-g3.2; LeafArea 1-g3.3; Internode length 1-g2.3; Stem determination 1-g1; Stem determination 1-g1; Interbranch length 1-g1.2; Stem termination type 2-g1; Lodging 3-g1; PH 6-g25; PH 3-g5; PH 1-g25; Node number 1-g2.2; PH 5-g3.2; PH 3-g1; LeafWidth 1-g5.3; PH 3-g2; PH 3-g6; Lodging 1-g1.3; Stem shape, main 1-g2.3; DTM9-g5; Total seed number 3-g1.1; DFTM 4-g18; DFTM 2-g18; Seed set 1-g50.2; Total seed number 3-g1.2; PH 3-g7 |
| 2.26; 2.27 | 20 | 35.621 | | Seed set1-g53.1; Shoot P 1-g35 |

* *Seed Met*: seed methionine content*; DFTM*: reproductive period duration; *Seed set*: seeds per pod; *SDS*: SDS resistance; *SCN*: resistance to SCN; *PROT*: seed protein content; *Sclero*: resistance to *S. sclerotiorum*; *WUE*: water use efficiency; *PubDen*: pubescence density; *SW*: seed weight; *AMIN*: seed amino acid content; *Shoot P*: shoot phosphorous content.
