# Supplementary figures and images for: Genetic Diversity and Selection Footprints in the Genome of Brazilian Soybean Cultivars
Source: Front Plant Sci. 2022 Mar 30;13:842571. doi: 10.3389/fpls.2022.842571 (PMC9006619; doi:10.3389/fpls.2022.842571)

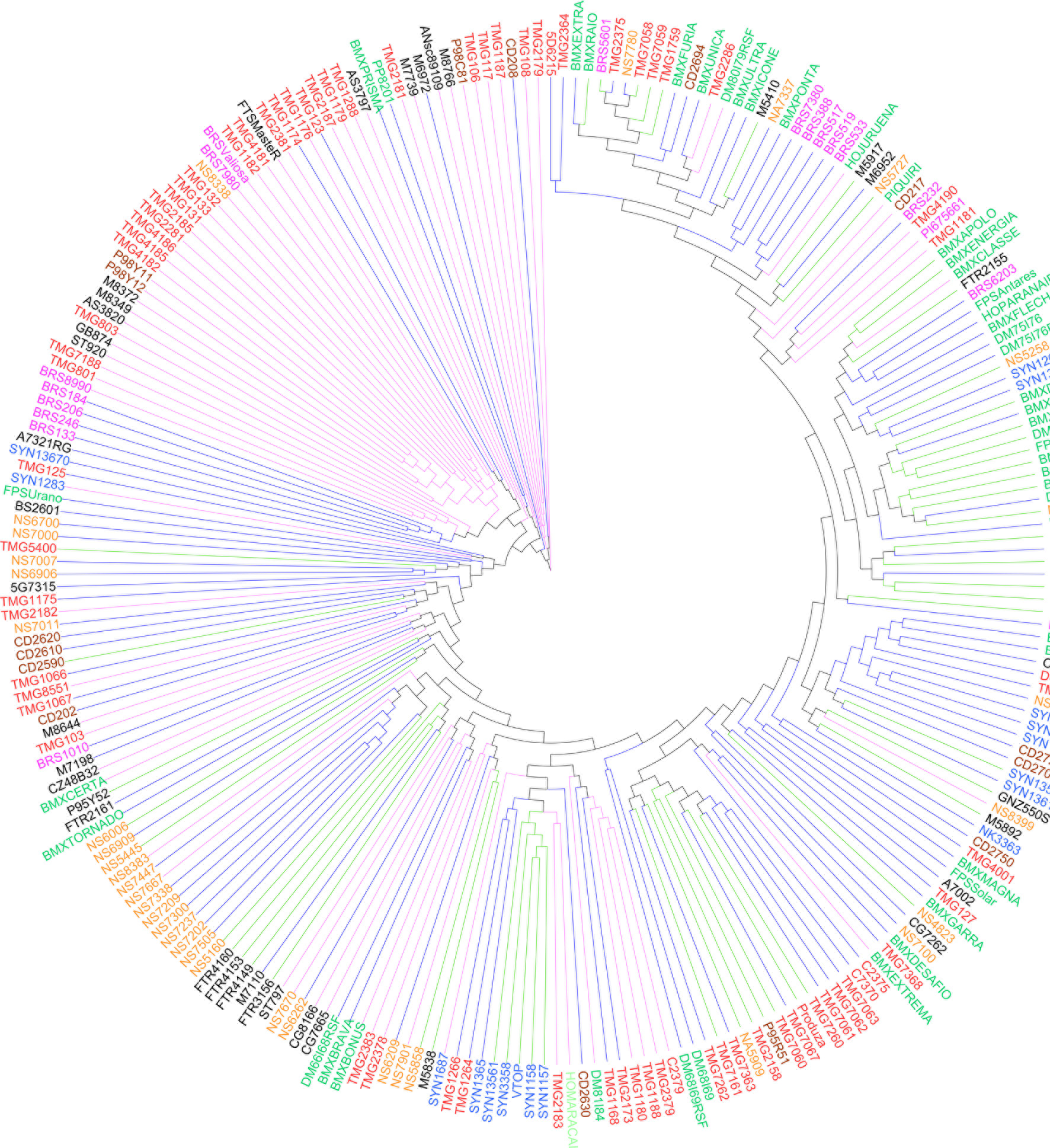

Supplement: Supplementary Figure 1 — Cluster analysis using the UPGMA method on genetic distances based on IBS among 247 Brazilian soybean cultivars, branches are colored by RMG whereas Cultivar labels are colored based on company of origin. [file Image_1.TIF]
